# Supplementary material for: Modeling glioblastoma heterogeneity as a dynamic network of cell states
Source: Mol Syst Biol. 2021 Sep 16;17(9):e10105. doi: 10.15252/msb.202010105 (PMC8444284; doi:10.15252/msb.202010105)
Supplement: Supplementary file 5 — Source Data for Figure 3 [file MSB-17-e10105-s001.zip › Figure3A_sourcedata/GSEA_3065/hallmarks_state3.GseaPreranked.1623416274744/HALLMARK_KRAS_SIGNALING_DN.html]

Details for gene set HALLMARK\_KRAS\_SIGNALING\_DN[GSEA]

|  || Dataset | state3 |
| Phenotype | NoPhenotypeAvailable |
| Upregulated in class | na\_neg |
| GeneSet | HALLMARK\_KRAS\_SIGNALING\_DN |
| Enrichment Score (ES) | -0.23059842 |
| Normalized Enrichment Score (NES) | -0.6700485 |
| Nominal p-value | 0.91681415 |
| FDR q-value | 1.0 |
| FWER p-Value | 1.0 |
Table: GSEA Results Summary

  

Fig 1: Enrichment plot: HALLMARK\_KRAS\_SIGNALING\_DN      
 Profile of the Running ES Score & Positions of GeneSet Members on the Rank Ordered List

  

| PROBE | GENE SYMBOL | GENE\_TITLE | RANK IN GENE LIST | RANK METRIC SCORE | RUNNING ES | CORE ENRICHMENT || 1 | IGFBP2 |  |  | 164 | 0.303 | 0.1190 | No |
| 2 | FGFR3 |  |  | 708 | 0.169 | 0.1334 | No |
| 3 | CELSR2 |  |  | 1135 | 0.127 | 0.1423 | No |
| 4 | VPS50 |  |  | 2075 | 0.073 | 0.0678 | No |
| 5 | SPHK2 |  |  | 2329 | 0.063 | 0.0675 | No |
| 6 | BARD1 |  |  | 2514 | 0.056 | 0.0721 | No |
| 7 | PTPRJ |  |  | 2571 | 0.054 | 0.0904 | No |
| 8 | SHOX2 |  |  | 2699 | 0.050 | 0.0986 | No |
| 9 | KMT2D |  |  | 2980 | 0.041 | 0.0853 | No |
| 10 | MFSD6 |  |  | 3104 | 0.038 | 0.0885 | No |
| 11 | CDKAL1 |  |  | 3451 | 0.029 | 0.0621 | No |
| 12 | SLC38A3 |  |  | 3682 | 0.023 | 0.0463 | No |
| 13 | BTG2 |  |  | 4070 | 0.013 | 0.0079 | No |
| 14 | CAMK1D |  |  | 4175 | 0.010 | 0.0007 | No |
| 15 | TGFB2 |  |  | 4673 | -0.003 | -0.0549 | No |
| 16 | LFNG |  |  | 4861 | -0.007 | -0.0729 | No |
| 17 | YPEL1 |  |  | 4935 | -0.009 | -0.0771 | No |
| 18 | SKIL |  |  | 5063 | -0.012 | -0.0860 | No |
| 19 | TCF7L1 |  |  | 5378 | -0.020 | -0.1128 | No |
| 20 | GTF3C5 |  |  | 5697 | -0.030 | -0.1357 | No |
| 21 | KCNQ2 |  |  | 5701 | -0.030 | -0.1224 | No |
| 22 | SNN |  |  | 5791 | -0.032 | -0.1179 | No |
| 23 | NUDT11 |  |  | 6775 | -0.070 | -0.1989 | Yes |
| 24 | EFHD1 |  |  | 6810 | -0.071 | -0.1704 | Yes |
| 25 | COPZ2 |  |  | 6894 | -0.076 | -0.1456 | Yes |
| 26 | CHST2 |  |  | 7170 | -0.092 | -0.1355 | Yes |
| 27 | PDK2 |  |  | 7397 | -0.108 | -0.1124 | Yes |
| 28 | KLHDC8A |  |  | 7407 | -0.109 | -0.0641 | Yes |
| 29 | SLC25A23 |  |  | 7505 | -0.118 | -0.0218 | Yes |
| 30 | CCDC106 |  |  | 7586 | -0.126 | 0.0263 | Yes |
| 31 | GAMT |  |  | 8235 | -0.236 | 0.0592 | Yes |
Table: GSEA details [plain text format]

  

Fig 2: HALLMARK\_KRAS\_SIGNALING\_DN: Random ES distribution      
 Gene set null distribution of ES for **HALLMARK\_KRAS\_SIGNALING\_DN**
